# Structural empowerment of triage nurses in the redirection of low-acuity patients in a Swiss emergency department: a mixed-methods convergent pilot study

**DOI:** 10.1186/s12873-026-01562-3

**Published:** 2026-04-02

**Authors:** Youcef Guechi, Yoann Noire, Thomas Castelain, Romain D’Agostino, Vincent Ribordy, Anne-Laure Feral-Pierssens

**Affiliations:** 1 Department of Emergency Medicine, Fribourg Cantonal Hospital, 2-6 Chemin des Pensionnats, Villars-sur-Glâne, 1752 Switzerland; 2 Faculty of Science and Medicine, University of Fribourg, 20 Avenue de l’Europe, Fribourg, 1700 Switzerland; 3 Laboratory for Education and Health Promotion, Paris-Sorbonne University, 74 Rue Marcel Cachin, Bobigny, 93017 France; 4 Emergency Department, Hôpital Avicenne, Assistance Publique-Hôpitaux de Paris, 125 Rue de Stalingrad, Bobigny, 93000 France

**Keywords:** Emergency department, Empowerment, Patient redirection, Switzerland, Triage nurse

## Abstract

**Background:**

Similar to most high-income countries, almost 30% of consultations in Swiss hospital emergency departments (EDs) involve patients with low-severity levels. Redirecting patients with low-severity conditions to non-hospital facilities has proven effective internationally in reducing ED overcrowding, wait times, and improving patient satisfaction without compromising safety. Specifically, this study aims to understand the conditions under which the patient redirection role is exercised in triage.

**Methods:**

We conducted a mixed method study among ED triage nurses (*n* = 23) in an urban teaching hospital in Western Switzerland. Data collection combined non-participant observation and semi-structured interviews. Data were analyzed through a systematic coding process where common themes across the studies were identified.

**Results:**

Structural empowerment assessed using the Conditions of Work Effectiveness Questionnaire-II evaluated six dimensions on a 5-point Likert scale (opportunities, access to information, organizational support, access to resources, formal power, informal power). Quantitative results showed high scores for opportunities and informal power, while access to information and organizational support was significantly lower. These trends converged with the qualitative data, with triage nurses describing real autonomy, but constrained by a lack of clinical feedback and variable interprofessional support.

**Conclusions:**

Although exploratory, our findings highlight the importance of organizational initiatives focusing on feedback loops, interprofessional support and continuous learning processes. Future research should transition from cross-sectional studies to longitudinal designs to assess the temporal impact of organizational changes, such as implementing formal feedback mechanisms on structural empowerment levels.

**Supplementary Information:**

The online version contains supplementary material available at 10.1186/s12873-026-01562-3.

## Background

In Switzerland, hospital emergency departments (EDs) receive more than 1.5 million patients each year, with an estimated annual increase of 3% to 5% since 2015 [1]. In 2013, nearly 30% of consultations involved patients with low severity levels, while the average waiting time before an initial medical contact sometimes exceeded several hours for this population [2, 3]. This phenomenon, common to most high-income countries, is amplified by staff shortages, saturated hospital capacity and increasingly complex needs [4]. It compromises the ability to provide rapid, safe and appropriate care to each patient and requires the exploration of innovative organizational solutions.

Among these solutions, redirecting patients with low-severity conditions to alternative care facilities has successfully reduced waiting times for initial medical contact and maintained or improved patient satisfaction across various healthcare systems, including Australia [5], Canada [6] or the Netherlands [7]. A recent meta-analysis [8] further supports these findings, suggesting that such models can mitigate ED overcrowding without negatively impacting clinical safety. Inspired by these experiences, some Swiss EDs have entrusted triage nurses with the decision to redirect patients, based on predefined protocols [9].

This transfer of responsibilities represents a significant change. It is no longer just a matter of assessing and directing, but also taking clinical decisions [10] with implications in terms of responsibility, safety and professional recognition. It is precisely at this level that Kanter’s theory of ‘structural empowerment’ provides a relevant framework for analysis [11]. Indeed, the autonomous exercise of new responsibilities depends not only on individual skills or protocols, but also on the structural conditions that enable triage and orientation nurses to act effectively, such as access to information, resources, training, organizational support and development opportunities [11]. Structural empowerment, already associated with greater job satisfaction, perceived quality of care and reduced burnout [12, 13], helps to understand the extent to which triage nurses have the necessary framework to take on this expanded role. However, this dimension remains largely unexplored, particularly in the Swiss context, and especially among nurses involved in triage [14]. The aim of this study was to explore the structural empowerment of triage nurses participating in a reorientation program in a Swiss ED, to identify weaknesses and guide future quality improvement processes.

## Methods

### Overview

This research is based on a pragmatic paradigm particularly well-suited to complex clinical settings, such as EDs. This approach justifies the integration of both qualitative and quantitative methods to address an applied issue, i.e., understanding the conditions under which the patient redirection role is exercised in triage. The mixed convergent parallel design enables the simultaneous quantitative measurement of structural empowerment and the qualitative exploration of the organizational and experiential factors that shape this experience so as to explore their influence on the quality, safety and effectiveness of care [15, 16]. The two components were conducted independently and with equal methodological priority to allow for an analytical comparison of the results. The integration was carried out at the interpretation stage to produce a comprehensive, contextualized and actionable understanding of the reorientation role entrusted to triage nurses [17].

The reporting of our qualitative component adheres to the Consolidated Criteria for Reporting Qualitative Studies (COREQ) checklist (Appendix A) [18] and the methodological quality of the overall mixed design was assessed using the Mixed Methods Appraisal Tool (Appendix B) [19].

### Study setting

This study was conducted in the emergency department of a Swiss urban teaching hospital serving a population of 350,000 people in the Swiss canton of Fribourg, covering approximately 1,671 km². It is the largest hospital in the region, and the only emergency department. It receives approximately 42,000 patients per year in all cases combined, while the overall LOS for emergency patients is approximately 440 min, and about 75% of patients are ambulatory cases. Triage nurses carrying out the triage process are specialized emergency care experts who have completed a two-year advanced training program following their standard three-year nursing degree [20].

Patients are triaged using the Swiss Emergency Triage Scale, a four-level severity scale based on the main presenting complaint, vital signs and medical history that exists since 1997 [21]. Level 1 refers to life-threatening emergencies requiring immediate medical attention. Level 2 concerns conditions requiring medical assessment within 20 min. Level 3 represents emergencies that can wait up to 120 min. Level 4 refers to relatively minor conditions whose treatment can be postponed. In 2024, a redirection system was put in place to enable triage nurses to redirect patients with low-severity conditions to medical centers and general practitioners’ offices via dedicated software [9].

### Quantitative analysis: standardized assessment of empowerment

Structural empowerment was assessed using the Conditions of Work Effectiveness Questionnaire-II (CWEQ-II) [22], which evaluates six dimensions on a 5-point Likert scale (Appendix C). This tool is well-established in nursing research [23] and presents excellent reliability, with previously reported Cronbach’s alpha coefficients ranging from 0.80 to 0.90 across its subscales [12, 24]. Of note, this questionnaire also includes a section containing two items dedicated to global empowerment that were not used in this study.

Following agreement from the scale’s original design team (Western University, Ontario, Canada), the English CWEQ-II was translated and cross-culturally adapted into French (Appendix D) based on Beaton’s methodology [25]. The process included two independent forward translations (English to French), followed by a consensus synthesis carried out by a third translator. This preliminary version was then pre-tested with a sample of the target population for cognitive debriefing, leading to minor rewording to ensure cultural appropriateness, conceptual equivalence, and optimal understandability before data collection.

Quantitative data were collected between May and November 2024. To be eligible for inclusion, participants had to be actively working as triage nurses and possess a minimum of six months of experience in the triage role at the time of data collection. This corresponds to systematic inclusion over a defined period. This choice was made to ensure a sufficient level of experience in the position and effective exposure to the triage system. Recruitment was therefore exhaustive and all eligible triage nurses were invited to participate by email.

The main criterion for evaluation was the score obtained on the CWEQ-II. Descriptive analyses (mean, standard deviation [SD], median, quartiles) were performed in order to characterize the levels and their dispersion according to the sub-dimensions of the questionnaire using IBM SPSS (version 29). The normality of the distribution of scores was assessed using the Shapiro–Wilk test. All sub-dimensions showed a normal distribution (*p* > 0.05), with the exception of organizational support (*p* = 0.005). Results were expressed as the mean ± SD for normal distributions and the median (interquartile range [IQR]) for non-normal distributions. Inter-individual variability was estimated using the coefficient of variation (CV = SD/mean) for normal data and a robust CV (robust CV = IQR/median) for non-normal data.

### Qualitative analysis: re-situated semi-structured interviews

We employed a targeted ethnography approach to explore the perceptions and experiences of triage nurses regarding the new patient redirection function within a real-world context. This methodological approach is particularly suited for understanding a specific phenomenon over a short period of time and is adapted to complex environments such as ED triage [26], with the analysis guided by Kanter’s theory of structural empowerment [11].

Data collection combined non-participant observation and semi-structured interviews with triage nurses. Patients who were triaged and potentially eligible for referral were identified by the triage nurse based on the reason for visiting the ED, age and visual appearance. Interviews were conducted in the ED within one hour of an actual patient redirection situation. During all these situations, the interviewer was present as a non-participating observer in order to provide a detailed description of the clinical scene. The researcher used “shadowing” [27], a technique that involves closely following an actor during their ordinary activities in order to capture practices in situ and decisions that are difficult to verbalize in an interview. During the interviews, the comments collected were contextualized using the scene description previously documented during the observation (covering the decisions, patient interactions and use of the software). All interviews were audio-recorded, fully transcribed and anonymized upon transcription. The interview guide covered the following dimensions: decision-making autonomy; organizational support; institutional recognition; role of the software; and practical feasibility conditions. Four pilot interviews were used to adjust the wording of the questions and refine the interview guide.

All interviews, including the four pilot interviews, were conducted solely by YN, a male nursing instructor and research nurse trained in qualitative methodology. While YN is an experienced former ED triage nurse, he is no longer employed in this department, providing him with contextual knowledge without the bias of being a current direct colleague. The decision was made to deliberately exclude the principal investigator (YG) from conducting the interviews due to their hierarchical status and in order to attenuate potential social desirability bias and promote a neutral environment for participants to share their experiences. To minimize the Hawthorne effect [28] during the triage nurse consultations, YN acted as a non-participating observer, remaining in the background and explicitly explaining the non-evaluative nature of the observation to the triage nurses.

Data analysis began with an initial vertical reading to allow the researchers (YN and RD) to immerse themselves in the interviews. A subsequent horizontal reading then enriched the list of codes using an inductive approach. To ensure explicit reflexivity and rigor, the coding process involved the same two researchers. Independent double coding was performed for 100% of the verbatim transcripts using the Atlas© (Atlas.ti 24.1.1 for Windows, Scientific Software Development GmbH, Berlin, Germany) software. Regular discussions were conducted between both researchers to limit the influence of preconceptions.

Interviews were conducted until data saturation was reached. Saturation was assessed sequentially and considered achieved when no new codes or themes emerged during the last two consecutive interviews, which is consistent with methodological recommendations for focused ethnography [29]. Additional interviews were conducted with triage nurses from the same ED outside of working hours in order to ensure that no new themes emerged.

### Data integration

**Fig. 1:**
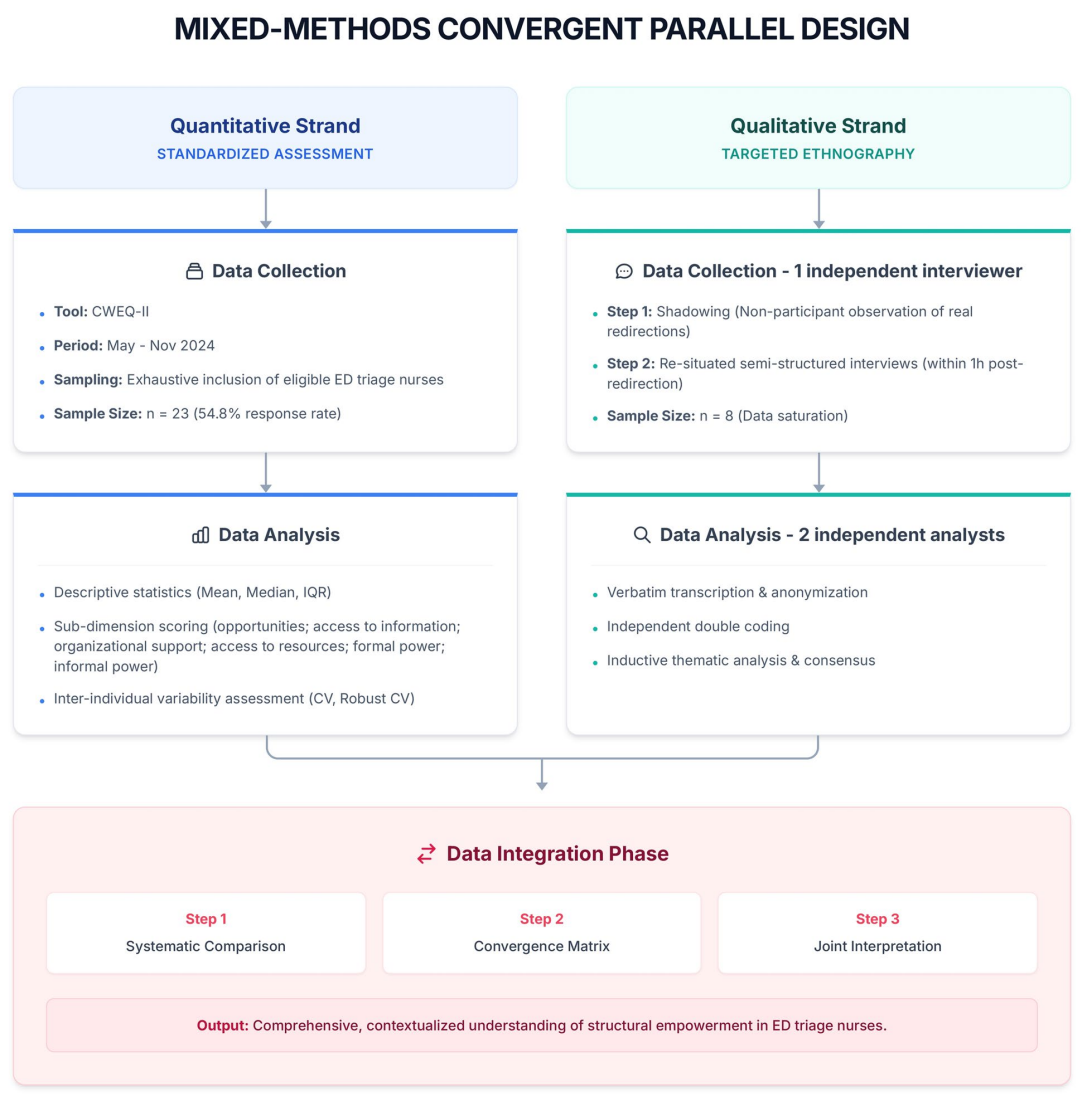
Conceptual model of data collection and integration

The integration of results followed three steps: systematic comparison of quantitative and qualitative results; development of a convergence matrix by two researchers (YN, YG); and joint interpretation of convergences, divergences and complementarities. The different steps of study design are illustrated in Fig. 1.

## Results

### Participant characteristics

The demographic and professional characteristics of participating triage nurses are summarized in Table 1. ED seniority varied widely, reflecting diverse career paths and varying levels of exposure to the role of triaging.

**Table 1:** Participant characteristics

Characteristics	*N* (%) or median [IQR]
Response rate	23/42 (54.8%)
Gender	
Female	15 (65%)
Male	8 (35%)
Time since graduation (mean)	5 years
Novices (< 5 years)	7 (30%)
Intermediate (5–10 years)	9 (39%)
Experienced (> 10 years)	7 (30%)
Number of hospital departments	3 [2–5]

IQR: interquartile range

### Structural empowerment scores (CWEQ-II)

**Fig. 2:**
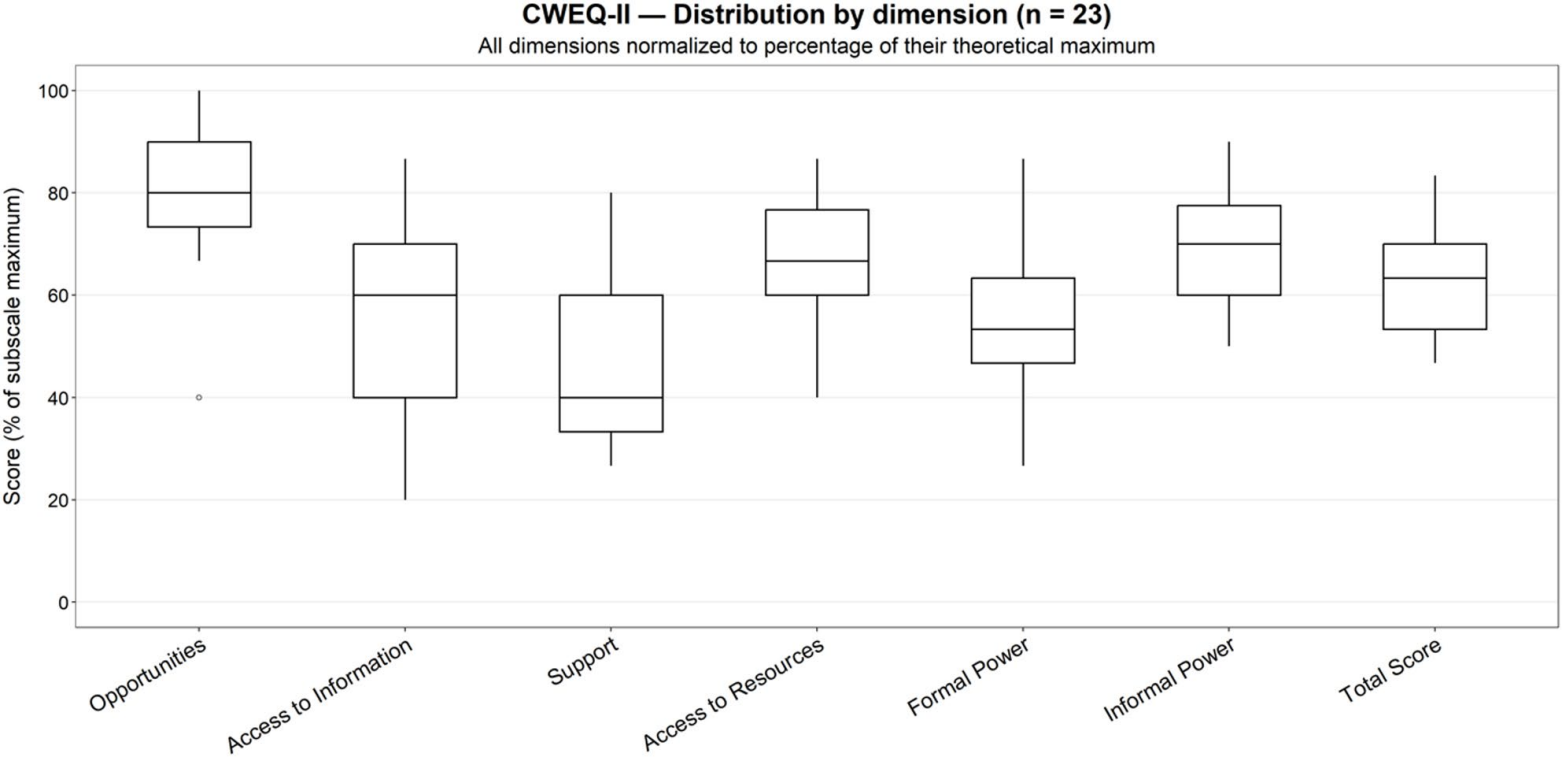
Distribution of CWEQ-II scores by dimension among triage nurses (*n* = 23). Each boxplot displays the median, interquartile range, and variability of individual responses. Scores were normalized to the maximum possible value for each dimension (opportunities, access to information, support, access to resources, formal power, informal power and total score) to allow a direct comparison on a 0–100% scale

The distribution of CWEQ-II scores by dimension among triage nurses (*n* = 23) is shown in Fig. 2 and descriptive statistics of structural empowerment scores in Table 2.

**Table 2:** Descriptive statistics of structural empowerment scores (CWEQ-II) among triage nurses (*n* = 23)

Sub-dimension	Mean ± SD	Median [IQR]	Minimum–maximum
Opportunities	4.04 ± 0.70	4.00 [0.83]	2.00–5.00
Access to information	2.77 ± 0.96	3.00 [1.5]	1.00–4.33
Organizational support*	2.33 ± 0.93	2.00 [1.33]	1.33–4.00
Access to resources	3.32 ± 0.65	3.33 [0.83]	2.00–4.33
Formal power	2.67 ± 0.82	2.67 [0.83]	1.33–4.33
Informal power	3.45 ± 0.62	3.50 [0.88]	2.5–4.50

* Non-normal distribution (Shapiro–Wilk test, *p* = 0.005)

SD: standard deviation; IQR: interquartile range

The mean total score for structural empowerment was 18.58/30 (SD = 3.41; median = 19.08; IQR = 5.00). Extreme values ranged from 14 to 25. No participant had a low score (< 13/30), nineteen had a moderate score (14–22/30), and four had a high score (> 22/30). Interindividual variability was particularly high for *access to information* (CV = 35%) and *organizational support* (robust CV = 67%), indicating significant heterogeneity in perceptions in these areas. Conversely, *opportunities* scored consistently high (CV = 14%), suggesting a perceived equitable access to professional development opportunities. Overall, triage nurses reported moderate structural empowerment: high for *opportunities* and *informal power*, but lower and more heterogeneous for *access to information* and *organizational support*.

### Qualitative results

Eight semi-structured interviews of between 40 and 60 min were conducted immediately after situations of redirection by triage nurses. All were transcribed, anonymized and analyzed using an inductive thematic approach. Eight triage nurses (male, 3 [37.5%]) participated in this phase. This group represented a wide range of seniority, with experience in the ED ranging from 1 to 19 years (mean, 6.5 years) and specific triage experience varying from 3 months to 11 years (mean, 4 years). Several key dimensions emerged from these interviews.

**1. Decision-making autonomy and responsibility**

Triage nurses described a high degree of decision-making autonomy in triage, combined with a high level of clinical responsibility. The lack of systematic feedback sometimes led to professional uncertainty: “*It’s a choice you make*,* but you’re not always sure it was the right one… we don’t get any feedback.*” (Nurse 4, 3 months’ experience). This paradox of autonomy without feedback sometimes generates professional insecurity. Others explicitly mentioned the legal risk: “*The problem with redirection is the responsibility if something goes wrong*.” (Nurse 7, 11 years’ experience).

**2. Access to resources: support perceived as incomplete**

IT tools and triage protocols were recognized by triage nurses as a useful safeguard, but also as a constraint limiting adaptation to real clinical situations: *“The triage tool is good*,* but it is also very restrictive*,* for example*,* for low back pain without trauma”* (nurse 8, 2 years’ experience). Several participants highlighted that these criteria do not always reflect the diversity of situations encountered and that coordination with partner structures could be improved: *“We don’t know how many patients are waiting in the emergency rooms*,* so sometimes we refer them a bit blindly”* (nurse 5, 3 years’ experience).

These comments reflected a perceived lack of information resources and coordination, consistent with the lower quantitative scores observed on the “information” dimension of the CWEQ-II questionnaire.

**3. Support and interprofessional collaboration**

The quality of medical support was considered essential: *“When you’re starting out*,* you always seek confirmation from a doctor or colleague”* (nurse 1, 1 year’s experience). However, interindividual variability is noted as a source of frustration: *“Some colleagues easily reorient patients*,* while others prefer to settle them in directly; it varies greatly from one person to another”* (nurse 6, 4 years’ experience). This heterogeneity reflected a lack of collective alignment and organizational support that was perceived as uneven, consistent with the quantitative results observed in this area.

**4. Professional experience: a moderating factor**

Seniority had a significant impact on perceptions of safety and the ability to justify patients’ redirection. Experienced nurses expressed greater confidence: *“Over the years*,* you’ve gotten better at explaining to patients why you’re redirecting them*,* and it goes more smoothly”* (nurse 7, 11 years’ experience). Conversely, the most novice practitioners relied heavily on medical validation. This interindividual variability reinforced the idea of empowerment in the making, strongly linked to the professional trajectory.

**5. Emotional rewards and constraints**

The triage role was described as rewarding because it has an impact on patient care conditions: *“It’s an interesting role because you have a direct impact on the flow”* (nurse 8, 2 years’ experience). Refusals to be redirected were a significant source of emotional stress, particularly for novice triage nurses: *“When the patient refuses to be redirected*,* it’s difficult and it wears you down”* (nurse 2, 1 year’s experience). This contrast highlighted that perceived empowerment can coexist with significant emotional tensions.

**6. Safety and quality of care: between benefits and uncertainties**

Redirection is generally perceived as a lever for organizational fluidity and prioritization of life-threatening emergencies. However, concerns remain about actual safety: *“We don’t always know if redirected patients have really received appropriate care afterwards”* (nurse 3, 8 years’ experience). This lack of follow-up reinforced the feeling of “partial” empowerment, where responsibility was assumed without any guarantee of the outcome.

### Data integration

The integrated analysis showed a strong convergence between the results, enabling the qualitative data to provide deeper context to the quantitative scores (Table 3). The quantitative results showed high scores for opportunities and informal power, while access to information and organizational support were significantly lower.

The low quantitative score obtained for “access to information” (average = 2.77) is illustrated in the qualitative reports. Findings show that nurses do not necessarily lack general medical guidelines but rather suffer from a structural absence of specific clinical feedback regarding the outcomes of their referral decisions. This lack of specific information generates the professional insecurity highlighted in the qualitative results.

Similarly, the “collaboration and support” dimension clearly illustrates this analytical convergence. The significant inter-individual variability associated with a global low score observed quantitatively in the CWEQ-II support subscale (Robus CV = 67%) is further contextualized by the qualitative data. Interviews reveal that delegated autonomy is not strictly standardized at the institutional level but depends on nurse’s characteristics and interpersonal relationships with individual doctors, thereby creating unequal experience from one triage nurse to another.

A notable complementarity concerned the moderating effect of professional experience, which was absent from the questionnaire, but central to the interviews. Novices often sought validation and medical support, while more experienced nurses expressed greater confidence and a better ability to persuade patients.

However, there were significant differences between the two sections, notably regarding the “resources” dimension. Despite moderate-to-high scores, the interviews reported a perceived rigidity of protocols and incomplete access to information about partner structures. This suggested that the resources perceived in the questionnaire only partially reflected the organizational constraints experienced in practice, raising the question of whether such resources really act as facilitators.

Overall, integrating the two components reinforced the consistency of the results and highlighted differentiated structural empowerment: high in decision-making autonomy and opportunities, but limited by a lack of structural feedback mechanisms and standardized interprofessional support.

**Table 3:** Convergence matrix of quantitative and qualitative results on structural empowerment dimensions

CWEQ-II dimension	Quantitative results (average scores)	Qualitative results (themes and selected verbatim quotes)	Integration (convergence/divergence)
Opportunity	High average score, reflecting perceived opportunities for influence	Experienced nurses feel valued for their impact on patient flow (“*It’s an interesting role*,* because you have a direct impact on the flow*”) Nurse 8	**Convergence: high scores aligned with narratives about opportunities**
Information	Lower average score, limited access to information and feedback	Lack of feedback perceived as stressful (“*It’s a choice you make*,* but you’re not always sure it was the right one*”) Nurse 4	**Convergence: low scores confirmed by narratives about lack of information**
Support	Lowest average score, high variability in perceived support	Support varies depending on colleagues and physicians (“*Some colleagues easily refer patients elsewhere*,* others prefer to admit them directly*”) Nurse 8	**Convergence: low support reflected in qualitative variability**
Resources	Moderate-to-high scores, but with contextual variability	Redirection assistance protocols perceived as useful but restrictive (“*The software helps*,* but it’s also very restrictive*”) Nurse 5	**Divergence: moderate-to-high scores contrasting with a qualitative discourse emphasizing rigidity**
Formal power	Low scores, reflecting the lack of formal authority associated with the triage role in the institution	The triage role is perceived as a responsibility with legal implications (“*The problem with referral is the responsibility if something goes wrong*”) Nurse 6	**Convergence: low quantitative scores enriched by concerns related to responsibility**
Informal power	Moderate-to-high scores, reflecting influence related to teamwork	Collaboration and informal exchanges shape decision-making and build trust (*“With years of experience*,* you know how to better explain to patients why you are referring them”)* Nurses 2 and 7	**Convergence: informal influence confirmed by accounts of peer support**

## Discussion

In this study, the quantitative results of the CWEQ-II indicated high scores for opportunities and decision-making autonomy, but lower scores for information and support. The interviews confirmed these trends, highlighting both growing confidence with experience and limitations related to lack of feedback, interprofessional heterogeneity and organizational constraints.

Our results are consistent with Laschinger’s seminal work, which demonstrated that access to resources, information and support is critical to satisfaction, retention and burnout reduction among nurses [12, 30]. Similarly, a recent review confirms the link between structural empowerment, organizational commitment and quality of care [13].

In the context of emergency services, several studies have shown that empowerment contributes to the quality of nursing decision-making and safety of care [31, 32]. Our qualitative data confirm these observations by highlighting that experienced triage nurses make greater use of their informal power (interprofessional collaboration, persuasion of patients), while younger nurses seek validation and medical support. This contrast reflects the importance of seniority and support, already observed in the literature on the development of clinical reasoning in triage [33]. Another point of convergence concerns the interindividual variability highlighted by the dispersion of scores and by the interviews. This variability can influence the consistency of referral decisions, echoing the concerns of several authors about the heterogeneity of triage practices and its impact on quality of care [34, 35].

### Implication

Our results suggest that structural empowerment in nurse-led redirection programs could be strengthened by several actionable strategies. First, institutions should establish regular multidisciplinary meetings involving triage nurses, nursing management, and physicians to promote case debriefings on complex cases and provide direct feedback on patient outcomes. Second, training strategies, both initials and continuous, should be updated to incorporate diverse, targeted teaching methods that enhance nurses’ confidence and clinical reasoning. Third, any triage software or decision-support tools must be designed and periodically updated to increase clinical applicability, ensuring they support nurse autonomy rather than acting as a rigid constraint. Finally, organizing discussion sessions with redirected patients would help highlight patients’ experiences and perspectives by fostering constructive dialogue between both parties. Implementing these targeted measures provides a solid foundation prior to further studies on the empowerment level of triage nurses and its objective clinical outcomes.

These levers are part of the dynamic of a learning organization where clinical practice becomes a continuous source of improvement and training. They are in line with recommendations advocating for work environments that promote autonomy, interprofessional collaboration and safety of care [12, 13].

### Strengths and limitations

The strengths of this study include the combined use of a validated instrument (CWEQ-II) and in-depth interviews, providing an integrated and contextualized view of structural empowerment.

However, the study has several limitations. First, this is a single-center study conducted in Switzerland, which limits the generalizability of the results. Second, the sample size, although appropriate for the exploratory and pilot nature of this mixed approach, remains too small for subgroup analyses (e.g. based on seniority). Third, the cross-sectional nature of the data collection does not allow for an assessment of the temporal effects of organizational change on the level of perceived empowerment. Fourth, while the interviewer (YN) possessed in-depth contextual knowledge as a former nurse experienced in triage, the fact that he is no longer part of the ED staff helped mitigate the risk of self-censorship that could occur when interviewing direct colleagues. Nevertheless, his prior expertise may have created a confirmation bias during both the observation and the initial coding phase.

Another limit stands in the response rate of our quantitative phase with only about half of the eligible nurses (23/42) answering. This introduces a potential non-response bias, as nurses who were either too busy, less interested, or experiencing higher levels of professional exhaustion may have been less likely to participate. While this could theoretically skew the empowerment scores, the high inter-individual variability observed in our results suggests that a wide spectrum of perceptions was nonetheless captured. Furthermore, the qualitative phase helped mitigate this limitation by exploring the structural constraints experienced by a diverse subset of nurses.

Although our study did not directly measure patient-centered outcomes, the literature in emergency medicine has shown that the organization and quality of triage influence the safety of care, particularly through classification errors and delays in treatment [36]. Strengthening the structural empowerment of triage nurses may support more consistent decision-making processes and could potentially reduce safety vulnerabilities during the redirection process, although this relationship should be confirmed by studies assessing patient-level outcomes. It could also have an organizational impact by promoting a smooth patient flow and appropriate referrals, and ultimately a human impact by contributing to well-being at work, team retention, and professional skills development. Therefore, subsequent studies must investigate how this nurse empowerment translates into clinical efficacy, patient safety, and successful redirection rates.

## Conclusion

This mixed-methods study revealed a differentiated structural empowerment among triage nurses. It is robust concerning decision-making autonomy and access to opportunities, yet critically limited by a lack of timely clinical feedback, consistent organizational support and access to information, These findings suggest a form of partial empowerment, where responsibility may be delegated without consistently providing the structural resources necessary to support decision-making. Although exploratory, our findings highlight the importance of organizational initiatives focusing on feedback loops, interprofessional support and continuous learning processes. Future research should transition from cross-sectional studies to longitudinal designs to assess the temporal impact of organizational changes, such as implementing formal feedback mechanisms on structural empowerment levels.

Additionally, as this model of out-of-hospital redirection expands to other institutions, large-scale multicenter studies will be highly valuable to explore how different organizational contexts influence the structural empowerment of triage nurses.

## Supplementary Information

Below is the link to the electronic supplementary material.

Supplementary Material 1

## Data Availability

The datasets generated and analyzed during this study are not publicly available due to the confidential nature of the qualitative interviews, but they are available upon reasonable request to the corresponding author.

## Abbreviations

COREQ: Consolidated criteria for reporting qualitative studies
CWEQ-II: Conditions of Work Effectiveness Questionnaire-II
CV: Coefficient of variation
ED: Emergency department
IQR: Interquartile range
SD: Standard deviation
