# Supplementary material for: Structural empowerment of triage nurses in the redirection of low-acuity patients in a Swiss emergency department: a mixed-methods convergent pilot study
Source: BMC Emerg Med. 2026 Apr 2;26:141. doi: 10.1186/s12873-026-01562-3 (PMC13169605; doi:10.1186/s12873-026-01562-3)
Supplement: Supplementary file 1 — Supplementary Material 1 [file 12873_2026_1562_MOESM1_ESM.docx]

**Appendix A**

Consolidated criteria for reporting qualitative studies (COREQ): 32-item checklist

Developed from:

Tong A, Sainsbury P, Craig J. Consolidated criteria for reporting qualitative research (COREQ): a 32-item checklist for interviews and focus groups. International Journal for Quality in Health Care. 2007. Volume 19, Number 6: pp. 349 – 357

| **Item No** | | **Guide Questions/Description** | **Reported on Page #** |  |
| --- | --- | --- | --- | --- |
| **Domain 1: Research team and reflexivity** | | | |  |
| **Personal Characteristics** | | | |  |
| 1. Interviewer/ facilitator | | Which author/s conducted the interview or focus group? | 7 |  |
| 2. Credentials | | What were the researcher’s credentials? E.g., PhD, MD | N/A |  |
| 3. Occupation | | What was their occupation at the time of the study? | 7-8 |  |
| 4. Gender | | Was the researcher male or female? | 7-8 |  |
| 5. Experience and training | | What experience or training did the researcher have? | 7-8 |  |
| **Relationship with participants** | | | |  |
| 6. Relationship established | | Was a relationship established prior to study commencement? | 8 |  |
| 7. Participant knowledge of the interviewer | | What did the participants know about the researcher? e.g. personal goals, reasons for doing the research? | 8 |  |
| 8. Interviewer characteristics | | What characteristics were reported about the interviewer/facilitator? e.g. Bias, assumptions, reasons and interests in the research topic | 8, 18 |  |
| **Domain 2: study design** | | |  |  |
| **Theoretical framework** | | |  |  |
| 9. Methodological orientation and Theory | What methodological orientation was stated to underpin the study? e.g. grounded theory, discourse analysis, ethnography, phenomenology, content analysis | 7 |  |  |
| **Participant selection** | | |  |  |
| 10. Sampling | How were participants selected? e.g., purposive, convenience, consecutive, snowball | 7-8 |  |  |
| 11. Method of approach | How were participants approached? e.g., face-to-face, telephone, mail, email | 7-8 |  |  |
| 12. Sample size | How many participants were in the study? | 11 |  |  |
| 13. Non-participation Setting | How many people refused to participate or dropped out? Reasons? | N/A |  |  |
| 14. Setting of data collection | Where was the data collected? e.g., home, clinic, workplace | 7 |  |  |
| 15. Presence of nonparticipants | Was anyone else present besides the participants and researchers? | 8 |  |  |
| 16. Description of sample | What are the important characteristics of the sample? e.g. demographic data, date | 11 |  |  |
| **Data collection** | | |  | No |
| 17. Interview guide | Were questions, prompts, and guides provided by the authors? Was it pilot tested? | 8 |  |  |
| 18. Repeat interviews | Were repeat interviews carried out? If yes, how many? | N/A |  |  |
| 19. Audio/visual recording | Did the research use audio or visual recording to collect the data? | 8 |  |  |
| 20. Field notes | Were field notes made during and/or after the interview or focus group? | 7-8 |  |  |
| 21. Duration | What was the duration of the interviews or focus group? | 11 |  |  |
| 22. Data saturation | Was data saturation discussed? | 8 |  |  |
| 23. Transcripts returned | Were transcripts returned to participants for comment and/or correction? | N/A |  |  |
| **Domain 3: analysis and findings** | | |  |  |
| **Data analysis** | | |  |  |
| 24. Number of data coders | How many data coders coded the data? | 8 |  |  |
| 25. Description of the coding tree | Did the authors provide a description of the coding tree? | N/A |  |  |
| 26. Derivation of themes | Were themes identified in advance or derived from the data? | 11 |  |  |
| 27. Software | What software, if applicable, was used to manage the data? | 8 |  |  |
| 28. Participant checking | Did participants provide feedback on the findings? | N/A |  |  |
| **Reporting** | | |  |  |
| 29. Quotations presented | Were participant quotations presented to illustrate the themes/findings? Was each quotation identified? e.g., participant number | 11-13 |  |  |
| 30. Data and findings consistent | Was there consistency between the data presented and the findings? | 14-15 |  |  |
| 31. Clarity of major themes | Were major themes clearly presented in the findings? | 11-13 |  |  |
| 32. Clarity of minor themes | Is there a description of diverse cases or a discussion of minor themes? | N/A |  |  |

**Appendix B**

**Part I: Mixed Methods Appraisal Tool, version 2018**

| **Category of study designs** | **Methodological quality criteria** | **Responses** | | | |
| --- | --- | --- | --- | --- | --- |
|  |  | Yes | No | Can’t tell | Comments |
| Screening questions  (for all types) | S1. Are there clear research questions? | Yes |  |  | The aim is to explore the structural empowerment of triage nurses participating in a reorientation program in a Swiss emergency department |
|  | S2. Do the collected data allow to address the research questions? | Yes |  |  | The study combines quantitative and qualitative data collection |
|  | *Further appraisal may not be feasible or appropriate when the answer is ‘No’ or ‘Can’t tell’ to one or both screening questions.* | | | | |
| 1. Qualitative | 1.1. Is the qualitative approach appropriate to answer the research question? | Yes |  |  | Targeted ethnography is suitable for understanding perceptions and experiences in a complex environment like triage |
|  | 1.2. Are the qualitative data collection methods adequate to address the research question? | Yes |  |  |  |
|  | 1.3. Are the findings adequately derived from the data? | Yes |  |  | Analysis used an inductive approach, and double coding was performed by two researchers. Data saturation was reached |
|  | 1.4. Is the interpretation of results sufficiently substantiated by data? | Yes |  |  | Findings (themes) are illustrated using participant quotations (verbatim). |
|  | 1.5. Is there coherence between qualitative data sources, collection, analysis and interpretation? | Yes |  |  |  |
| 2. Quantitative randomized controlled trials | 2.1. Is randomization appropriately performed? |  |  |  |  |
|  | 2.2. Are the groups comparable at baseline? |  |  |  |  |
|  | 2.3. Are there complete outcome data? |  |  |  |  |
|  | 2.4. Are outcome assessors blinded to the intervention provided? |  |  |  |  |
|  | 2.5 Did the participants adhere to the assigned intervention? |  |  |  |  |
| 3. Quantitative non-randomized | 3.1. Are the participants representative of the target population? |  |  |  |  |
|  | 3.2. Are measurements appropriate regarding both the outcome and intervention (or exposure)? |  |  |  |  |
|  | 3.3. Are there complete outcome data? |  |  |  |  |
|  | 3.4. Are the confounders accounted for in the design and analysis? |  |  |  |  |
|  | 3.5. During the study period, is the intervention administered (or exposure occurred) as intended? |  |  |  |  |
| 4. Quantitative descriptive | 4.1. Is the sampling strategy relevant to address the research question? | Yes |  |  | Recruitment was exhaustive of all triage nurses who had worked for more than six months |
|  | 4.2. Is the sample representative of the target population? | Yes |  |  | Recruitment was exhaustive within the department to characterize levels and dispersion of CWEQ-II dimensions, fitting the descriptive objective. |
|  | 4.3. Are the measurements appropriate? | Yes |  |  | The validated Conditions of Work Effectiveness Questionnaire-II (CWEQ-II) was used. The French version was translated and adapted following established methodology (Beaton's) |
|  | 4.4. Is the risk of nonresponse bias low? |  |  | Can’t tell |  |
|  | 4.5. Is the statistical analysis appropriate to answer the research question? | Yes |  |  | Descriptive analyses (mean, SD, median, IQR) were performed, which is appropriate as the objective was descriptive. |
| 5. Mixed methods | 5.1. Is there an adequate rationale for using a mixed methods design to address the research question? | Yes |  |  | The design was chosen to study simultaneously the level of perceived empowerment (quantitative) and the mechanisms that shape this experience (qualitative), addressing the complexity of the phenomenon. |
|  | 5.2. Are the different components of the study effectively integrated to answer the research question? | Yes |  |  | Integration followed three steps: systematic comparison, development of a convergence matrix (Table 2), and joint interpretation of convergences, divergences, and complementarities |
|  | 5.3. Are the outputs of the integration of qualitative and quantitative components adequately interpreted? | Yes |  |  |  |
|  | 5.4. Are divergences and inconsistencies between quantitative and qualitative results adequately addressed? | Yes |  |  | The discrepancy in the "Resources" dimension (moderate/high scores vs. perceived protocol rigidity/incomplete information) is explicitly discussed and interpreted |
|  | 5.5. Do the different components of the study adhere to the quality criteria of each tradition of the methods involved? |  |  | Can’t tell |  |

**Appendix C**

CONDITIONS FOR WORK EFFECTIVENESS QUESTIONNAIRE-II

| **How much of each kind of opportunity do you have in your present job?** | | | | |
| --- | --- | --- | --- | --- |
| 1 = None | 2 | 3 = Some | 4 | 5 = A Lot |

| 1. Challenging work | 1 | 2 | 3 | 4 | 5 |
| --- | --- | --- | --- | --- | --- |
| 1. The chance to gain new skills and knowledge on the job | 1 | 2 | 3 | 4 | 5 |
| 1. Tasks that use all of your own skills and knowledge | 1 | 2 | 3 | 4 | 5 |
|  | | | | | |

| **How much access to information do you have in your present job?** | | | | |
| --- | --- | --- | --- | --- |
| 1 = No Knowledge | 2 | 3 = Some Knowledge | 4 | 5 = Know A Lot |

| 1. The current state of the hospital | 1 | 2 | 3 | 4 | 5 |
| --- | --- | --- | --- | --- | --- |
| 1. The values of top management | 1 | 2 | 3 | 4 | 5 |
| 1. The goals of top management | 1 | 2 | 3 | 4 | 5 |
|  | | | | | |

| **How much access to support do you have in your present job?** | | | | |
| --- | --- | --- | --- | --- |
| 1 = None | 2 | 3 = Some | 4 | 5 = A Lot |

| 1. Specific information about things you do well | 1 | 2 | 3 | 4 | 5 |
| --- | --- | --- | --- | --- | --- |
| 1. Specific comments about things you could improve | 1 | 2 | 3 | 4 | 5 |
| 1. Helpful hints or problem solving advice | 1 | 2 | 3 | 4 | 5 |
|  | | | | | |

| **How much access to resources do you have in your present job?** | | | | |
| --- | --- | --- | --- | --- |
| 1 = None | 2 | 3 = Some | 4 | 5 = A Lot |

| 1. Time available to do necessary paperwork | 1 | 2 | 3 | 4 | 5 |
| --- | --- | --- | --- | --- | --- |
| 1. Time available to accomplish job requirements | 1 | 2 | 3 | 4 | 5 |
| 1. Acquiring temporary help when needed | 1 | 2 | 3 | 4 | 5 |
|  | | | | | |

| **In my work setting/job: (JAS)** | | | | |
| --- | --- | --- | --- | --- |
| 1 = None | 2 | 3 = Some | 4 | 5 = A Lot |

| 1. the rewards for innovation on the job are | 1 | 2 | 3 | 4 | 5 |
| --- | --- | --- | --- | --- | --- |
| 1. the amount of flexibility in my job is | 1 | 2 | 3 | 4 | 5 |
| 1. the amount of visibility of my work-related activities within the institution is | 1 | 2 | 3 | 4 | 5 |
|  | | | | | |

| **How much opportunity do you have for these activities in your present job: (ORS)** | | | | |
| --- | --- | --- | --- | --- |
| 1 = None | 2 | 3 = Some | 4 | 5 = A Lot |

| 1. Collaborating on patient care with physicians | 1 | 2 | 3 | 4 | 5 |
| --- | --- | --- | --- | --- | --- |
| 1. Being sought out by peers for help with problems | 1 | 2 | 3 | 4 | 5 |
| 1. Being sought out by managers for help with problems | 1 | 2 | 3 | 4 | 5 |
| 1. Seeking out ideas from professionals other than physicians, e.g., physiotherapists, occupational therapists, dieticians | 1 | 2 | 3 | 4 | 5 |

GLOBAL EMPOWERMENT

| **How much of each kind of opportunity do you have in your present job?** | | | | |
| --- | --- | --- | --- | --- |
| 1 = Strongly Disagree | 2 | 3 | 4 | 5 = Strongly Agree |

| 1. Overall, my current work environment empowers me to accomplish my work in an effective manner | 1 | 2 | 3 | 4 | 5 |
| --- | --- | --- | --- | --- | --- |
| 1. Overall, I consider my workplace to be an empowering environment | 1 | 2 | 3 | 4 | 5 |

**Appendix D**

**Participant Questionnaire – Français**

| **Opportunités** | | | | |
| --- | --- | --- | --- | --- |
| 1= Pas du tout | 2 = Dans une faible mesure | 3 = Neutre | 4 = Dans une large mesure | 5 = Absolument |

| 1. Votre travail est-il stimulant? | 1 | 2 | 3 | 4 | 5 |
| --- | --- | --- | --- | --- | --- |
| 1. Votre travail vous offre-t-il l’opportunité d’acquérir de nouvelles compétences et de nouveaux savoirs ? | 1 | 2 | 3 | 4 | 5 |
| 1. Votre travail présente-t-il des tâches qui nécessitent l’utilisation de vos propres compétences et savoirs ? | 1 | 2 | 3 | 4 | 5 |
|  | | | | | |

| **Accès à l’information** |
| --- |
| \| 1= Pas du tout \| 2 = Dans une faible mesure \| 3 = Neutre \| 4 = Dans une large mesure \| 5 = Absolument \| \| --- \| --- \| --- \| --- \| --- \| |

| 1. Avez-vous connaissance d’informations sur l’état actuel de l’hôpital ? | 1 | 2 | 3 | 4 | 5 |
| --- | --- | --- | --- | --- | --- |
| 1. Avez-vous connaissance d’informations sur les valeurs de l’établissement ? | 1 | 2 | 3 | 4 | 5 |
| 1. Avez-vous connaissance d’informations sur les objectifs de la hiérarchie ? | 1 | 2 | 3 | 4 | 5 |
|  | | | | | |

| **Support organisationnel** | | | | |
| --- | --- | --- | --- | --- |
| 1= Pas du tout | 2 = Dans une faible mesure | 3 = Neutre | 4 = Dans une large mesure | 5 = Absolument |

| 1. Recevez-vous des informations spécifiques sur ce que vous faîtes bien ? | 1 | 2 | 3 | 4 | 5 |
| --- | --- | --- | --- | --- | --- |
| 1. Recevez-vous des informations spécifiques sur les choses que vous pourriez améliorer ? | 1 | 2 | 3 | 4 | 5 |
| 1. Recevez-vous des conseils pour la résolution de problèmes ? | 1 | 2 | 3 | 4 | 5 |
|  | | | | | |

| **Accès aux ressources** | | | | |
| --- | --- | --- | --- | --- |
| 1= Pas du tout | 2 = Dans une faible mesure | 3 = Neutre | 4 = Dans une large mesure | 5 = Absolument |

| 1. Avez-vous du temps disponible pour effectuer les démarches administratives nécessaires ? | 1 | 2 | 3 | 4 | 5 |
| --- | --- | --- | --- | --- | --- |
| 1. Avez-vous le temps de réaliser votre travail ? | 1 | 2 | 3 | 4 | 5 |
| 1. Avez-vous l’occasion d’obtenir de l’aide temporairement lorsque cela est nécessaire ? | 1 | 2 | 3 | 4 | 5 |
|  | | | | | |

| **Environnement de travail** | | | | |
| --- | --- | --- | --- | --- |
| 1= Pas du tout | 2 = Dans une faible mesure | 3 = Neutre | 4 = Dans une large mesure | 5 = Absolument |

| 1. Les innovations au travail sont-t-elles récompensées ? | 1 | 2 | 3 | 4 | 5 |
| --- | --- | --- | --- | --- | --- |
| 1. Mon emploi est-il fléxible ? | 1 | 2 | 3 | 4 | 5 |
| 1. Mes activités professionnelles sont-elles visibles au sein de l’institution ? | 1 | 2 | 3 | 4 | 5 |
|  | | | | | |

| **Autres activités** | | | | |
| --- | --- | --- | --- | --- |
| 1= Pas du tout | 2 = Dans une faible mesure | 3 = Neutre | 4 = Dans une large mesure | 5 = Absolument |

| 1. Mon travail me permet-il de collaborer avec les médecins pour la prise en charge des patients ? | 1 | 2 | 3 | 4 | 5 |
| --- | --- | --- | --- | --- | --- |
| 1. Mon travail permet-il que mes pairs me sollicitent pour obtenir de l’aide en cas de problème ? | 1 | 2 | 3 | 4 | 5 |
| 1. Mon travail me permet-il d’être sollicité par les cadres pour obtenir de l’aide en cas de problème ? | 1 | 2 | 3 | 4 | 5 |
|  | | | | | |

**DONNEES SOCIODEMOGRAPHIQUES**

1. Êtes-vous un homme ou une femme ?
2. Dans quelle tranche d’âge vous situez-vous ? 20-30 ans / 30-40 ans / 40-50 ans / 50-60 ans / 60-70 ans
3. Depuis combien de temps travaillez-vous dans votre établissement actuel ? Entre 0 et 5 ans / entre 5 et 10 ans / Entre 10 et 20 ans / Entre 20 et 30 ans
4. En quelle année avez-vous obtenu votre diplôme d’infirmier?
5. En quelle année avez-vous obtenu votre diplôme d’infirmier expert en soins d’urgence ?
6. Depuis l’obtention de votre diplôme d’infirmier,

- dans combien d’établissements avez-vous exercé ?
- dans combien de service ?
- et dans combien de service urgence ?
